# Assessment of Chronic Disease Epidemiology Workforce in State Health Departments — United States, 2003

**Published:** 2007-06-15

**Authors:** Paul Z Siegel, Sara L Huston, Kenneth E Powell, Mark S Baptiste, Hugh H Tilson, Jillian Jacobellis, Ross C Brownson

**Affiliations:** Division of Adult and Community Health, National Center for Chronic Disease Prevention and Health Promotion, Centers for Disease Control and Prevention, Atlanta, Ga; North Carolina Division of Public Health, Raleigh, NC; Atlanta, Ga; New York State Department of Health, Albany, NY; Public Health Leadership Program, University of North Carolina, School of Public Health, Chapel Hill, NC; Prevention Services Division, Colorado Department of Public Health and Environment, Denver, Colo; Prevention Research Center, Saint Louis University School of Public Health, St. Louis, Mo

## To the Editor:

Despite the huge burden of chronic disease in the United States — four of every five deaths and $325 billion in health care costs and lost worker productivity per year (1) — the number of epidemiologists who work on chronic disease at state health departments remains less than half the number who work on infectious disease and less than one-third the combined number who work on infectious disease and bioterrorism (2). The percentage of state and territorial health departments that reported having "full/almost full or substantial" capacity in epidemiology and surveillance for chronic disease did not improve between 2001 (52%) and 2004 (48%) (3). The Council of State and Territorial Epidemiologists (CSTE) recommends that every state have a minimum of five full-time chronic disease epidemiologists (CDEs), at least one of whom should have a doctoral degree (4).

To obtain detailed information about chronic disease epidemiology capacity, including workforce, at state health departments, CSTE conducted a national assessment in March 2003. States were asked to report the number of people who spent at least 50% of their time at the health department doing work related to chronic disease epidemiology, as well as the training (academic degrees) and years of chronic disease epidemiology experience for each of those people. In the survey, chronic disease epidemiology was described as

[…analyzing and interpreting…] data related to chronic diseases or risk factors for chronic diseases. At the very least, chronic disease epidemiologists combine data from different sources, such as vital statistics and population estimates, to calculate rates. Commonly they calculate rates at one or across several points in time for groups of persons (e.g., rates by sex, rates by health district). Depending upon their duties and skills, [Behavioral Risk Factor Surveillance System] coordinators, cancer registry workers, people in data analyst positions, and others may be considered chronic disease epidemiologists. If they calculate and interpret rates, they may be counted as chronic disease epidemiologists.

States were asked to include CDEs who worked at the health department even if they received their paycheck from another organization (e.g., an academic institution).

Responses were received from 47 states (including the District of Columbia) during April through July 2003. One state is excluded from the analysis because of missing information about the educational background of chronic disease epidemiology staff. Among the 46 states included in the analysis, 25 (54%) had five or more full-time CDEs, as recommended by CSTE; 40 (87%) had at least one doctoral-level CDE; and 24 (52%) had both (Table).

Our analysis indicates that, despite the large public health burden of chronic diseases, as of 2003 only about half of states had the minimum chronic disease epidemiologic workforce recommended by CSTE. As the U.S. population continues to age, states will need even more CDEs to maintain adequate surveillance and plan data-based interventions to control high-prevalence chronic conditions such as heart disease, stroke, cancer, diabetes, chronic respiratory diseases, and arthritis, as well as risk factors such as smoking, physical inactivity, poor nutrition, and obesity.

State agencies can use the CSTE recommendations and the results of this survey to customize their approach to developing capacity for chronic disease epidemiology. For example, the 16 states that have a doctoral-level CDE but fewer than five total CDEs might focus their workforce development efforts on hiring junior-level epidemiologists. Every state should have an epidemiology job series in its personnel system (4) to facilitate the hiring of chronic disease and other types of epidemiologists. Results of this capacity survey can also help CSTE and the Centers for Disease Control and Prevention (CDC) identify states that are highest priority for technical assistance and capacity-building support.

Workforce, although critical, is not the only component of chronic disease epidemiology capacity. Other components of capacity that have been suggested include 1) access to data and consultants, 2) data analysis, 3) data interpretation, 4) information dissemination, and 5) outreach and partnership (4). To improve chronic disease epidemiology capacity in state health departments, CDC and CSTE should

- Develop capacity-building strategies to address specific gaps in either workforce or other components of capacity in state public health agencies.
- Develop minimum standards for routine analysis of chronic disease data.
- Identify factors that foster a productive chronic disease epidemiologic unit in state public health agencies.
- Identify elements of the 2003 survey that are most likely to be useful for ongoing surveillance of capacity for chronic disease epidemiology.

Additional previously published recommendations for increasing capacity should be given serious consideration as well. These include the following:

- Identify funding to support CDEs in states with the greatest need.
- Foster, support, and encourage collaboration among state health agencies and academic organizations in teaching, research, and special joint state and academic projects.
- Develop a national educational effort targeted at state health officials, agency or bureau directors, and program administrators to enhance understanding and awareness of the role of epidemiologists and chronic disease programs in states (5).

Our study is subject to at least three limitations. First, it may overestimate the chronic disease epidemiology workforce because all epidemiologists who work at least 50% of their time at the health department doing work related to chronic disease epidemiology were considered to be full-time employees. Therefore, the true number of chronic disease epidemiologists in some health departments may actually be lower than what is reported here. Second, five states were excluded from the analysis because of nonresponse or missing data. Third, some states, especially those with large populations or an excessive burden of chronic disease, may require more than the minimum workforce recommended by CSTE.

Although techniques for assessing and characterizing capacity for chronic disease epidemiology should be refined, some steps to increase workforce have been undertaken. Since 1991 the chronic disease State-based Epidemiology for Public Health Program Support (STEPPS) activity at CDC has provided staff or salary support to 30 states for chronic disease epidemiology positions. Of the 23 states that no longer receive support from STEPPS, at least 16 (70%) have made the successful transition to one or more chronic disease epidemiology positions that are supported independently by the state. More recent capacity-building activities include the CSTE/CDC Applied Epidemiology Fellowship Program (6), which places trainees under the supervision of experienced CDEs at state health departments, and a mentoring program, which pairs CDEs in states that have limited capacity with more experienced epidemiologists for a period of 6 to 12 months. Both of these activities met with early success but had limited implementation because of limited funding.

Our study did not examine factors that may be associated with the epidemiology capacity in state health departments. We intend to conduct such analysis in the future, which will consider factors such as workforce, competencies, access to key data sets and software, and academic linkages.

## Figures and Tables

**Table:** Number of States with Minimum Recommended Chronic Disease Epidemiologist (CDE)^a^ Workforce, United States, 2003

**State had at least one doctoral-level CDE^b^**	State had at least 5 CDEs		

	**Yes (%)**	**No (%)**	**Total (%)**
Yes	24 (52)	16 (35)	40 (87)
No	1 (2)	5 (11)	6 (13)
Total	25 (54)	21 (46)	46 (100)

a Any CDE (filled position, at least 50% of the person’s time is spent doing work related to chronic disease epidemiology), regardless of education or experience.

b CDE (filled position, at least 50% of the person’s time is spent doing work related to chronic disease epidemiology) with any doctoral degree (e.g., PhD, MD), regardless of experience.
